# Community-Acquired Methicillin-Resistant Pyogenic Liver Abscess: A Case Report

**DOI:** 10.1177/2324709616660576

**Published:** 2016-08-03

**Authors:** Joel Cherian, Rahul Singh, Muralidhar Varma, Sudha Vidyasagar, Chiranjay Mukhopadhyay

**Affiliations:** 1 Kasturba Medical College Manipal, Kasturba Hospital, Manipal, Karnataka, India

**Keywords:** pyogenic liver abscess, community-acquired infection, MRSA, methicillin-resistant *Staphylococcus aureus*

## Abstract

Pyogenic liver abscesses are rare with an incidence of 0.5% to 0.8% and are mostly due to hepatobiliary causes (40% to 60%). Most are polymicrobial with less than 10% being caused by *Staphylococcus aureus*. Of these, few are caused by methicillin-resistant *Staphylococcus aureus* (MRSA) and fewer still by a community-acquired strain. Here we present a case study of a patient with a community-acquired MRSA liver abscess. The patient presented with fever since 1 month and tender hepatomegaly. Blood tests revealed elevated levels of alkaline phosphatase, C-reactive protein, erythrocyte sedimentation rate, and neutrophilic leukocytosis. Blood cultures were sterile. Ultrasound of the abdomen showed multiple abscesses, from which pus was drained and MRSA isolated. Computed tomography of the abdomen did not show any source of infection, and an amebic serology was negative. The patient was started on vancomycin for 2 weeks, following which he became afebrile and was discharged on oral linezolid for 4 more weeks. Normally a liver abscess is treated empirically with ceftriaxone for pyogenic liver abscess and metronidazole for amebic liver abscess. However, if the patient has risk factors for a *Staphylococcal* infection, it is imperative that antibiotics covering gram-positive organisms be added while waiting for culture reports.

## Introduction

Pyogenic liver abscesses are rare with an incidence of 0.5% to 0.8% and cause approximately 15 out of 100 000 hospital admissions.^1^ Pyogenic liver abscesses are generally due to hepatobiliary causes (40% to 60%) leading to infection and local spread. These include cholelithiasis, biliary strictures, or congenital anomalies. Other causes may include perforated bowel or appendicitis. Rarely hematogenous spread is seen as well. Most of these abscesses are polymicrobial in nature with less than 10% of cases caused by *Staphylococcus aureus*.^2^ Of these cases, few are caused by methicillin-resistant *Staphylococcus aureus* (MRSA) and fewer still by a community-acquired strain. At least 13 cases of pyogenic liver abscesses caused by MRSA are reported in English literature, of which a minimum of 5 are community acquired. These reports suggest that pyogenic liver abscesses of MRSA origin may follow a particularly aggressive course although variability of severity was also seen. Here we report a case of community-acquired MRSA liver abscess.

## Case Report

**Figure 1.**
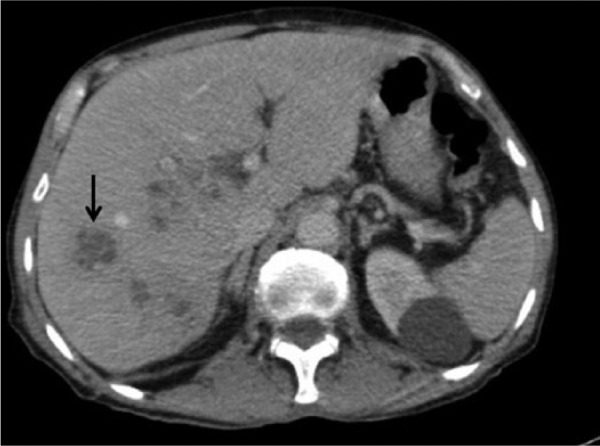
Contrast-enhanced computed tomography of the abdomen showing multiple liver abscesses with arrow pointing at largest abscess measuring 7.6 cm × 4.8 cm × 4.2 cm.

An 81-year-old male with no premorbidities presented with high-grade fever with chills and rigors since 1 month. He had presented previously to a local hospital for a skin infection and was treated empirically with clarithromycin for 2 months. He returned complaining of fever for which he was referred to our hospital. A physical examination found his vitals to be stable and his abdomen was soft with tender hepatomegaly. Blood tests revealed an elevated alkaline phosphatase level of 162 IU/L associated with neutrophilic leukocytosis (19.7 × 10^3^/L, neutrophils 79.2%), an elevated C-reactive protein level (301.1 mg/L), and an elevated erythrocyte sedimentation rate (58 mm/h). Blood cultures were sterile, and echocardiogram was normal. Ultrasound abdomen showed the presence of multiple liver abscesses, following which ultrasound-guided aspiration was done. Five milliliters of thick brownish pus aspirate isolated gram-positive cocci, found to be MRSA. Antibiogram revealed resistance to ciprofloxacin/ofloxacin, cloxacillin, erythromycin, and clindamycin. The isolate was sensitive to trimethoprim/sulfamethoxazole, gentamicin, tetracycline/doxycycline, linezolid, rifampicin, teicoplanin, and vancomycin. Contrast-enhanced computed tomography (CT) of the abdomen revealed multiple liver abscesses, the largest measuring 7.6 cm × 4.8 cm × 4.2 cm (arrow in Figure 1). CT abdomen did not, however, reveal any source of infection for the liver abscesses. An amebic serology was done and was negative as well. The patient became afebrile after 2 weeks of vancomycin, following which he was started on oral linezolid, which was continued for 4 more weeks. Follow-up after 3 months showed complete resolution of the abscess on ultrasonography, and the patient reported significant improvement in general condition.

## Discussion

The most common presentation of a liver abscess is fever, chills, and right upper quadrant pain with laboratory investigations showing low albumin, elevated alkaline phosphatase, and leukocytosis.^3^

In our patient, no intra-abdominal focus of infection was found and he had no recent hospital admissions, indicating that this MRSA infection was community acquired. We assumed that the source of infection was the skin since he was previously treated for a skin infection with antibiotics. The rarity of this case is 2-fold in that not only is it a pyogenic liver abscess caused by MRSA but also that the source is the community.

The mainstay of treatment of a liver abscess is drainage and appropriate systemic antibiotics.^4^ In the case of MRSA causing a liver abscess, the selection of an appropriate antibiotic is crucial. The first-line therapy for MRSA infection is intravenous vancomycin. Another viable option is linezolid, which seems to have the same, if not better, efficacy when compared to vancomycin.^5,6^ Oral and intravenous linezolid achieve the same plasma concentrations and there seems to be no difference in the occurrence of adverse effects.^7,8^ The average duration of treatment of a liver abscess is 4 to 6 weeks.^9^ While linezolid is bacteriostatic and vancomycin is bactericidal, oral preparations are an option with linezolid, but not with vancomycin. The use of an oral preparation allows for a reduction in hospital stay. In our case, we were able to send the patient home on oral linezolid for 4 weeks, reducing the hospital stay substantially. Normally a liver abscess is treated empirically with ceftriaxone for pyogenic liver abscess and metronidazole for amebic liver abscess. However, if the patient has risk factors for a *Staphylococcal* infection, it is imperative that antibiotics that cover gram-positive organisms be added while waiting for culture reports.

There have been instances where surgical intervention has been needed to treat a liver abscess, including those caused by MRSA.^10^ The indications for surgical intervention include no improvement after 4 to 7 days of percutaneous drainage; viscous pus in a thick-walled abscess; multiple, large, or loculated abscesses; and associated intra-abdominal surgical pathology.^11-13^
