# Erratum

**DOI:** 10.1177/2324709617693122

**Published:** 2017-02-01

## Abstract

Cherian J, Singh R, Varma M, Vidyasagar S, Mukhopadhyay C. Community-acquired methicillin-resistant pyogenic liver abscess: a case report. *J Investig Med High Impact Case Rep*. 2016;4(3):1-3. doi: 10.1177/2324709616660576

The authors Joel Cherian and Rahul Singh are medical students and should not have the degree “MD” against their names in the author by-line.

The correct author by-line is mentioned below.

Joel Cherian, Rahul Singh, Muralidhar Varma, MD, Sudha Vidyasagar, MD, and Chiranjay Mukhopadhyay, MD

SAGE regrets the error.

